# Socioeconomic vulnerability associated to *Toxoplasma gondii* exposure in southern Brazil

**DOI:** 10.1371/journal.pone.0212375

**Published:** 2019-02-14

**Authors:** Marcelle Mareze, Aline do Nascimento Benitez, Ana Pérola Drulla Brandão, Fernanda Pinto-Ferreira, Ana Carolina Miura, Felippe Danyel Cardoso Martins, Eloiza Teles Caldart, Alexander Welker Biondo, Roberta Lemos Freire, Regina Mitsuka-Breganó, Italmar Teodorico Navarro

**Affiliations:** 1 Department of Preventive Veterinary Medicine, Laboratory of Zoonoses and Public Health, Londrina State University, Londrina, Paraná, Brazil; 2 Department of Animal Production and Health, São Paulo State University, Araçatuba, São Paulo, Brazil; 3 Department of Preventive Veterinary Medicine, University of São Paulo, São Paulo, Brazil; 4 Department of Veterinary Medicine, Federal University of Paraná, Curitiba, Paraná Brazil; 5 Department of Comparative Pathobiology, Purdue University, West Lafayette, Indiana United States of America; Instituto Rene Rachou, BRAZIL

## Abstract

Human toxoplasmosis, a protozoonosis caused by *Toxoplasma gondii*, has been described as a worldwide foodborne disease with important public health impact. Despite infection has reportedly varied due to differences in alimentary, cultural and hygienic habits and geographic region, social vulnerability influence on toxoplasmosis distribution remains to be fully established. Accordingly, the present study has aimed to assess *T*. *gondii* seroprevalence and factors associated to social vulnerability for infection in households of Ivaiporã, southern Brazil, with 33.6% population making half minimum wage or less, ranked 1,055^th^ in population (31,816 habitants), 1,406^th^ in per capita income (U$ 211.80 per month) and 1,021^st^ in HDI (0.764) out of 5,570 Brazilian cities. Serum samples and epidemiological questionnaires were obtained from citizen volunteers with official City Secretary of Health assistance in 2015 and 2016. In overall, serosurvey has revealed 526/715 (73.57%) positive samples for anti-*T*. *gondii* antibodies by Indirect Fluorescent Antibody Test. Logistic regression has shown a significant increase associated to adults (p = 0.021) and elderly (p = 0.014) people, illiterates (p = 0.025), unemployment (p <0.001) and lack of household water tank (p = 0.039). On the other hand, sex (male or female), living area (urban or rural), yard hygiene, meat ingestion, sand or land contact, owning pets (dog, cat or both) were not significant variables of positivity for anti-*T*. *gondii* antibodies in the surveyed population. Although no significant spatial cluster was found, high intensity areas of seropositive individuals were located in the Kernel map where the suburban neighborhoods are located. In conclusion, socioeconomic vulnerability determinants may be associated to *Toxoplasma gondii* exposure. The increased risk due to illiteracy, adult or elderly age, unemployment and lack of household water tank were confirmed by multivariate analysis and the influence of low family income for seropositivity by the spatial analysis.

## Introduction

Human toxoplasmosis, a protozoonosis caused by intracellular parasite *Toxoplasma gondii*, has been described as a worldwide foodborne disease with important public health impact [1,2]. The foodborne transmission may occur by intake of shedding oocysts from felid feces and contaminate water, infecting a wide range of intermediate hosts including dogs and human beings [3]. Transmission occurs through consumption of vegetables contaminated with faecal oocysts, uncooked meat, fresh milk from acutely infected goats, trans-placental infection during pregnancy, organ transplantation or blood transfusion [4,5]. Cases of toxoplasmosis in immunocompetent patients has been mostly asymptomatic; in immunocompromised individuals, shown a tendency of more severe clinical manifestations. Pregnant women may be an important risk group, since vertical transmission may trigger reproductive disorders, abortion, congenital disease with unspecific systemic symptoms, neurological and severe ocular damage in fetuses, auditive impairment, psychomotor development, hyperactivity and attention deficit [6–9].

Prevalence of *T*. *gondii* seropositivity may vary worldwide from 10% to 90%, mainly due to regional variations [1], with lifetime persistence of infection, typically asymptomatic, potentially latent and associated to psychiatric disorders [10–12] or including death in immunocompromised patients [13–15]. Associated factors for toxoplasmosis have been shown relevance on *T*. *gondii* seroprevalence, including school level [16,17] and low family income in latent toxoplasmosis related to cognitive deficit [18].

Spatial analysis has been recently applied to epidemiologic investigation of affected individuals in urban and rural settings, providing a clear view of territory spreading and a better understanding of disease distribution. Identification of factors associated to disease in considered populations may contribute to extrapolation and development of effective prophylactic strategies [19,20].

Despite infection has reportedly varied due to differences in alimentary, cultural and hygienic habits and geographic region, social vulnerability influence on distribution remains to be fully established. Accordingly, the present study has aimed to assess *T*. *gondii* seroprevalence and factors associated to social vulnerability for infection in households of Ivaiporã, southern Brazil, 33.6% of its population, ranked 1,055^th^ in population (31,816 habitants), 1,406^th^ in per capita income (U$ 211.80 per month) and 1,021^st^ in HDI (0.764) out of 5,570 Brazilian cities.

## Materials and methods

The present study has been approved by the Ethics Committee of Research Involving Human Beings at the Londrina State University (protocol 1,177,975/2015) and conducted as part of the official activities coordinated by the City Secretary of Health. Consent was obtained by the signature of a Free Prior Informed Consent Form

**Fig 1:**
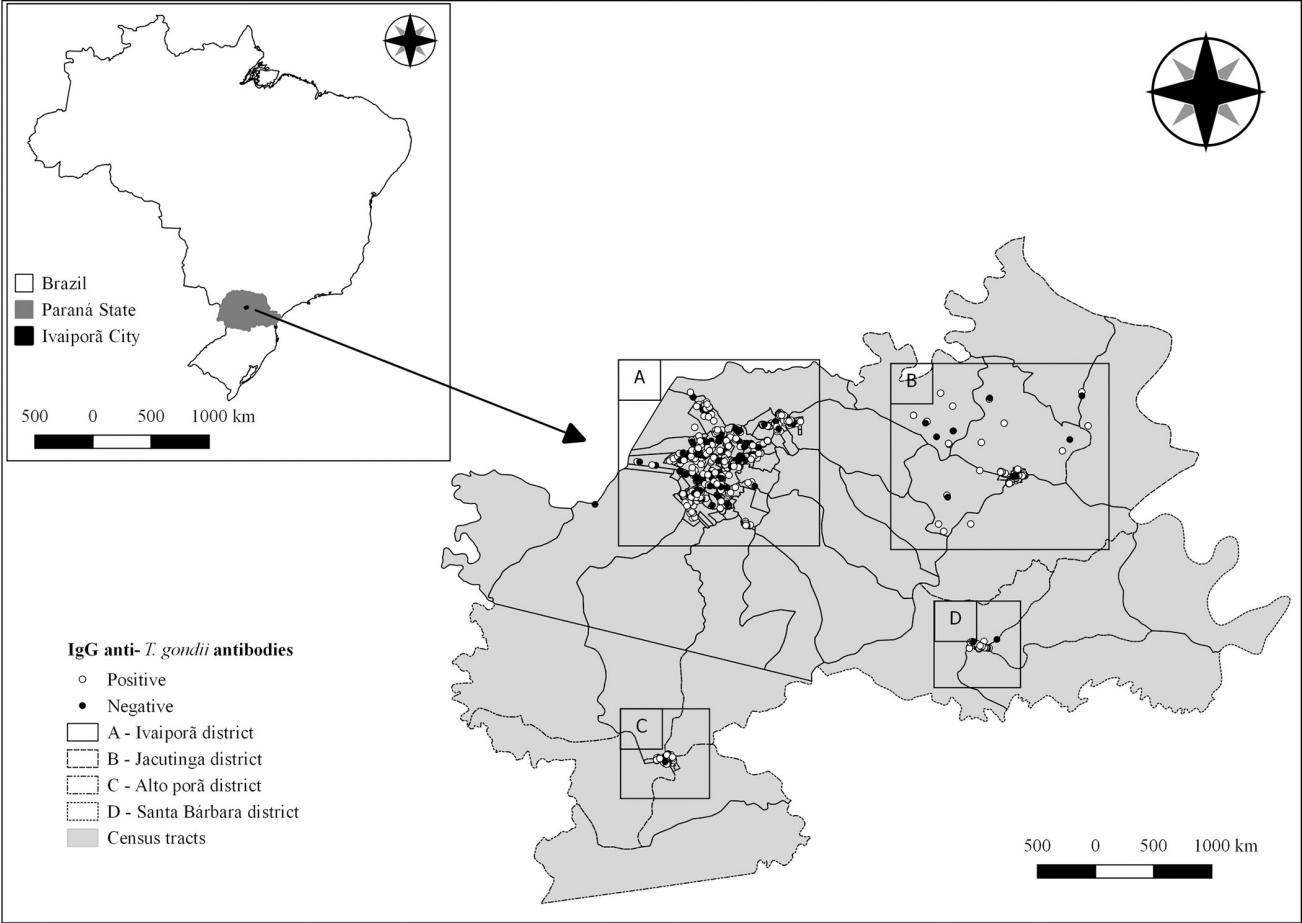
Location of Ivaiporã city, Paraná State, Brazil, including the serology results for IgG anti-T. gondii antibodies in 715 human samples tested by IFAT, from 2015 to 2016.

Ivaiporã city (24°14'52"S and 51°41'06"W), located in Paraná State, southern Brazil (Fig 1), composed of central area and districts of Jacutinga, Alto Porã and Santa Bárbara, has been characterized by distinct rural and urban areas. Situated within the Atlantic Forest biome with humid subtropical climate (Cfa), has historically presented an average pluviosity of 168 mm, 76% humidity and temperatures varying from 15°C to 26°C, [21]. The estimated population at the time of survey was 31,816 habitants (ranked 1,055^th^ in population out of 5,570 Brazilian cities), with majority of 27,438 (86.20%) people living in urban area.

Despite the city has established a public treated water system and presented relatively high (0.730) human development index (HDI) at the time of survey (ranked 1,021^st^ out of 5,570 Brazilian cities), Gini index related to economic level inequality was intermediate (0.4882) [21], and social vulnerability index (SVI) was classified as low (0.263) [22]. The State minimum wage at the time was of R$834.00 (U$ 243.15 at 3.43 exchange rate in 2016), with city ranked as 1,406^th^ in per capita income (U$ 211.80 per month), with 33.6% of the population with a monthly income of up to ½ minimum wage, out of 5,570 Brazilian cities.

Minimum sampling of 570 human beings was determined by the OpenEpi software [23], based on estimated population [21] and with an expected prevalence of 50%, confidence level of 95%, error of 5% and Deff of 1.5. A multidisciplinary taskforce conducted by the city hall and involving the Basic Units for Health (UBS) was organized and conducted throughout 2015 and 2016 in several regions of the city. This taskforce was announced through print media, electronic media and sound cars, inviting the entire population to participate. On the scheduled date for each region, immediately after a zoonosis prevention program, the taskforce professionals collected blood samples and applied questionnaires (S1 File) to all those who agreed to participate voluntarily. Therefore, the sampling of this study is considered a convenience sampling. Serum samples were voluntarily obtained and kept at -20° C until testing for IgG anti-*T gondii* antibodies by Indirect Fluorescent Antibody Test (IFAT), as previously described [24]. Tachyzoites of RH strain were used as antigens, a commercially available anti-antibody species-specific conjugated with fluorescein isothiocyanate was used (Sigma-Aldrich, Saint Louis, MO, USA). Negative and positive controls were added in each slide, the considered cut-off was 1:16, reactive samples in higher dilutions were considered positive.

A questionnaire containing volunteer information (age, sex, address) along with questions on volunteer habits and intra and extra-domiciliary environments was used as previously described [25], in addition to questions related to socioeconomical conditions, alimentary and sanitary habits, own pet relationship and epidemiological factor for *T*. *gondii* infection.

Data was gathered and analyzed by a free software package (R version 3.4.2, R Core Team, Vienna, Austria). First, a univariate analysis was performed to test variable association to seropositivity using the Q-square or Exact Fisher Tests, *Odds Ratio* (OR) calculation and Confidence Interval (CI) of OR, with 5% of confidence level (α). Following, a multivariate analysis was performed, including associated risk factors with p-value of < 0.020 in a logistic regression model. Interactions between independent variables were tested and included in the model.

The geographical points were obtained by spatial coordinates, based on the residential address given during questionnaire interview. A purely spatial analysis of cluster using Bernoulli model [26] was performed in each city region, using a commercial software (SaTScan^TM^ version 9.4.4, Boston, MA, USA) with a 5% significance level. A Kernel intensity map was applied for evaluation of *T*. *gondii* seropositivity distribution. All maps were constructed using a commercial software [27].

## Results

The sampled population was of 715 people, representing 2.25% of overall Ivaiporã City population. No clinical complaint related to disease was made at the time of survey. Serosurvey has revealed 526/715 (73.57%) positive samples for anti-*T*. *gondii* antibodies by Indirect Fluorescent Antibody Test. The resulting seroprevalence of 73.57% presented a 95% confidence interval (CI) between 70.33% and 76.80%. Considering only the woman of childbearing age (15 to 49 years old), 171/248 were positive, with a seroprevalence of 68.95% (95% CI: 65.56–72.34).

The volunteer profile was heterogeneous, with 530/715 (74.13%) living on urban area, 650/715 (90.91%) over 18-years old, 495/715 (69.23%) females, 486/715 (67.97%) with basic school level, 667/715 (93.29%) with low income (<3 minimal wage), 393/715 (54.97%) with some kind of occupation, 564/715 (78.88%) owning pets, of which 532/564 (94.33%) dog owners, 197/564 (34.93%) cat owners and 165/564 (29.26%) owning both and most people lacked household water tank (417/715, 58.32%) (Table 1).

**Table 1:** Results of univariate analysis of associated risk factors for seropositivity of IgG anti-*T*. *gondii* antibodies in 715 human samples tested by IFAT, from 2015 to 2016, in Ivaiporã city, Paraná State, Brazil.

Variable	Descriptive	Positive	OR	95% CI	p-value
	Yes/Total (%)	Yes/Total (%)			
**Gender**					
Male	220/715 (30.77)	165/220 (75.00)	0.898	0.62–1.29	0.562
Female	495/715 (69.23)	361/495 (72.93)			
**Age range**					
Young (< = 18 years old)	65/715 (9.09)	35/65 (53.85)	2.647	1.58–4.45	<0.001*
Adult (> 18 years old)	650/715 (90.91)	491/650 (75.54)			
**Schooling**					
Low (up to elementary school)	486/715 (67.97)	380/486 (78.19)	2.038	1.44–2.88	<0.001*
High (high school or higher education)	229/715 (32.03)	146/229 (63.76)			
**Monthly income**					
< = 3 minimum wage	667/715 (93.29)	497/667 (74.51)	1.915	1.05–3.51	0.035*
> 3 minimum wage	48/715 (6.71)	29/48 (60.42)			
**Occupation**					
No (retired, unemployed people and homemakers)	322/715 (45.03)	269/322 (83.54)	2.686	1.87–3.85	<0.001*
Yes	393/715 (54.97)	257/393 (65.39)			
**Area**					
Rural	48/715 (6.71)	35/48 (72.92)	0.965	0.50–1.87	0.916
Urban	667/715 (93.29)	491/667 (73.61)			
**Source of drinking water**					
Public system	662/715 (92.59)	488/662 (73.72)	0.903	0.49–1.68	0.749
Other	53/715 (7.41)	38/53 (71.70)			
**Presence of water tank**					
Yes	298/715 (41.68)	206/298 (69.13)	1.473	1.05–2.06	0.023*
No	417/715 (58.32)	320/417 (76.74)			
**Covers water tank**					
Yes	293/296 (98.99)	203/293 (69.28)	0.222	0.02–2.48	0.221
No	3/296 (1.01)	1/3 (33.33)			
**Cleans water tank**					
Yes	261/296 (88.18)	179/261 (68.58)	1.145	0.53–2.50	0.732
No	35/296 (11.82)	25/35 (71.43)			
**Cleaning frequency of water tank**					
At least once a year	202/296 (68.24)	137/202 (67.82)	1.177	0.69–2.01	0.550
Do not know or do not clean	94/296 (31.76)	67/94 (71.28)			
**Sewer system**					
Public system	59/714 (8.26)	41/59 (69.49)	1.243	0.70–2.22	0.464
Other	655/714 (91.74)	484/655 (73.89)			
**Garbage disposal**					
Correct	690/715 (96.50)	504/690 (73.04)	2.706	0.80–9.15	0.109*
Incorrect	25/715 (3.50)	22/25 (88.00)			
**Backyard**					
Clean	455/715 (63.64)	336/455 (73.85)	0.961	0.68–1.36	0.823
Dirty	260/715 (36.36)	190/260 (73.08)			
**Cleaning frequency of backyard**					
Low (fortnightly, monthly or occasionally	122/714 (17.09)	88/122 (72.13)	0.918	0.59–1.42	0.701
High (at least once a week)	592/714 (82.91)	437/592 (73.82)			
**Washes fruits and vegetables**					
Yes	707/715 (98.88)	520/707 (73.55)	1.079	0.22–5.39	0.926
No	8/715 (1.12)	6/8 (75.00)			
**Product used to wash fruits and vegetables**					
Just water	528/707 (74.68)	393/528 (74.43)	1.192	0.82–1.74	0.362
Sanitary water or vinegar	179/707 (25.32)	127/179 (70.95)			
**Washes hands prior to meals**					
Always	637/715 (89.09)	467/637 (73.31)	1.131	0.66–1.95	0.660
Sometimes or never	78/715 (10.91)	59/78 (75.64)			
**Meat consumption**					
Yes	709/715 (99.16)	522/709 (73.62)	1.396	0.25–7.68	0.702
No	6/715 (0.84)	4/6 (66.67)			
**Beef meat consumption**					
Yes	621/709 (87.59)	458/621 (73.75)	1.054	0.64–1.74	0.838
No	88/709 (12.41)	64/88 (72.73)			
**Pork meat consumption**					
Yes	596/709 (84.06)	440/596 (73.83)	1.066	0.68–1.68	0.781
No	113/709 (15.94)	82/113 (72.57)			
**Sheep meat consumption**					
Yes	177/708 (25.00)	128/177 (72.32)	0.917	0.63–1.34	0.658
No	531/708 (75.00)	393/531 (74.01)			
**Poultry meat consumption**					
Yes	646/709 (91.11)	473/646 (73.22)	0.781	0.42–1.45	0.434
No	63/709 (8.89)	49/63 (77.78)			
**Fish consumption**					
Yes	448/708 (63.28)	326/448 (72.77)	0.891	0.63–1.26	0.516
No	260/708 (36.72)	195/260 (75.00)			
**Raw or undercooked meat consumption**					
Yes	126/709 (17.77)	94/126 (74.60)	1.064	0.68–1.65	0.784
No	583/709 (82.23)	428/583 (73.41)			
**Raw kebab consumption**					
Yes	50/708 (7.06)	33/50 (66.00)	0.676	0.37–1.25	0.209
No	658/708 (92.94)	488/658 (74.16)			
**Barbecue undercooked meat consumption**					
Yes	190/709 (26.80)	142/190 (74.74)	1.082	0.74–1.58	0.684
No	519/709 (73.20)	380/519 (73.22)			
**Smoked sausage consumption**					
Yes	424/709 (59.80)	311/424 (73.35)	0.965	0.69–1.36	0.839
No	285/709 (40.20)	211/285 (74.04)			
**Fresh sausage consumption**					
Yes	506/709 (71.37)	375/506 (74.11)	1.091	0.76–1.57	0.643
No	203/709 (28.63)	147/203 (72.41)			
**Salami consumption**					
Yes	222/709 (31.31)	157/222 (70.72)	0.807	0.57–1.15	0.237
No	487/709 (68.69)	365/487 (74.95)			
**Raw milk**					
Yes	135/715 (18.88)	100/135 (74.07)	1.033	0.67–1.58	0.882
No	580/715 (81.12)	426/580 (73.45)			
**Sand/soil contact**					
Yes	453/715 (63.36)	343/453 (75.72)	1.346	0.96–1.89	0.087*
No	262/715 (36.64)	183/262 (69.85)			
**Own animals**					
Yes	564/715 (78.88)	420/564 (74.47)	1.238	0.83–1.84	0.291
No	151/715 (21.12)	106/151 (70.20)			
**Own dogs**					
Yes	532/564 (94.33)	397/532 (74.62)	1.151	0.52–2.55	0.729
No	32/564 (5.67)	23/32 (71.88)			
**Own cats**					
Yes	197/564 (34.93)	148/197 (75.13)	1.055	0.71–1.57	0.793
No	367/564 (65.07)	272/367 (74.11)			

p<0.05, Q-square or Exact Fisher Tests, OR: odds ratio, CI: Confidence Interval, MW: the monthly State Minimum Wage at the time of survey was R$834.00, equivalent to US$ 243.15 with an exchange rate of 3.43 in 2016 for US$ Dollar to R$ Real.

* Variables included in the logistic model.

In the univariate analysis, the risk factors associated with toxoplasmosis seroprevalence (p-value <0.05) included not having occupation outside home (retired or unemployed people and own housekeeper) (OR 2.69, CI: 1.87–3.85), older than 18 years (OR 2.65, CI: 1.58–4.45), none or low school level (OR 2.04 CI: 1.45–2.88), low income (OR 1.92, CI: 1.05–3.51) and lack of household water tank (OR 1.47, CI 1.05–2.06) (Table 1). Other analyzed risk factors such as sex (male or female), living area (urban or rural), yard hygiene, meat ingestion, contact with sand or land and owning pets (dog, cat or both) were not associated with seropositivity on surveyed population.

Multivariate analysis has shown a significant increased risk associated with unemployed people (OR 1.67, CI: 1.18–2.64), older than 18 years, adults (18 to 59 years old) (OR 2.60, 95% CI: 1.15–5.84) and elderly (over 60 years old) (OR 3.10, 95% CI: 1.26–7.67) people and lacking household water tank (OR 1.46; 95% CI:1.02–2.08). Regarding school level, only illiteracy was a significant risk factor (OR 4.37; 95% CI: 1,21–15,88) (Table 2). All other risk factors included in the model (income, garbage disposal, contact with sand or land and presence of bathroom, toilet and piped water in the household) were not statistically significant.

**Table 2:** Final logistic model for the analysis of risk factors associate for seropositivity of IgG anti-*T*. *gondii* antibodies in 715 human samples tested by IFAT, from 2015 to 2016, in the city of Ivaiporã, Paraná State, Brazil.

Variables	Adjusted OR	CI	p-value
**Age range** (Ref = child up to 11 years old)			
Adolescent (12 to 18 years old)	1.67	0.6–4.68	0.330
Adult (19 to 59 years old)	2.60	1.15–5.84	0.021*
Elderly (above 60 years old)	3.10	1.26–7.67	0.014*
**Schooling** (Ref = higher education)			
High School	0.80	0.38–1.69	0.558
Elementary School	1.24	0.59–2.61	0.573
Illiterate	4.37	1.21–15.88	0.025*
**Occupation** (retired, unemployed people and homemakers)	1.67	1.18–2.64	0.006*
**Household lacking water tank**	1.46	1.02–2.08	0.039*

OR: odds ratio, CI: Confidence Interval.

* Statistically significant variables

**Fig 2:**
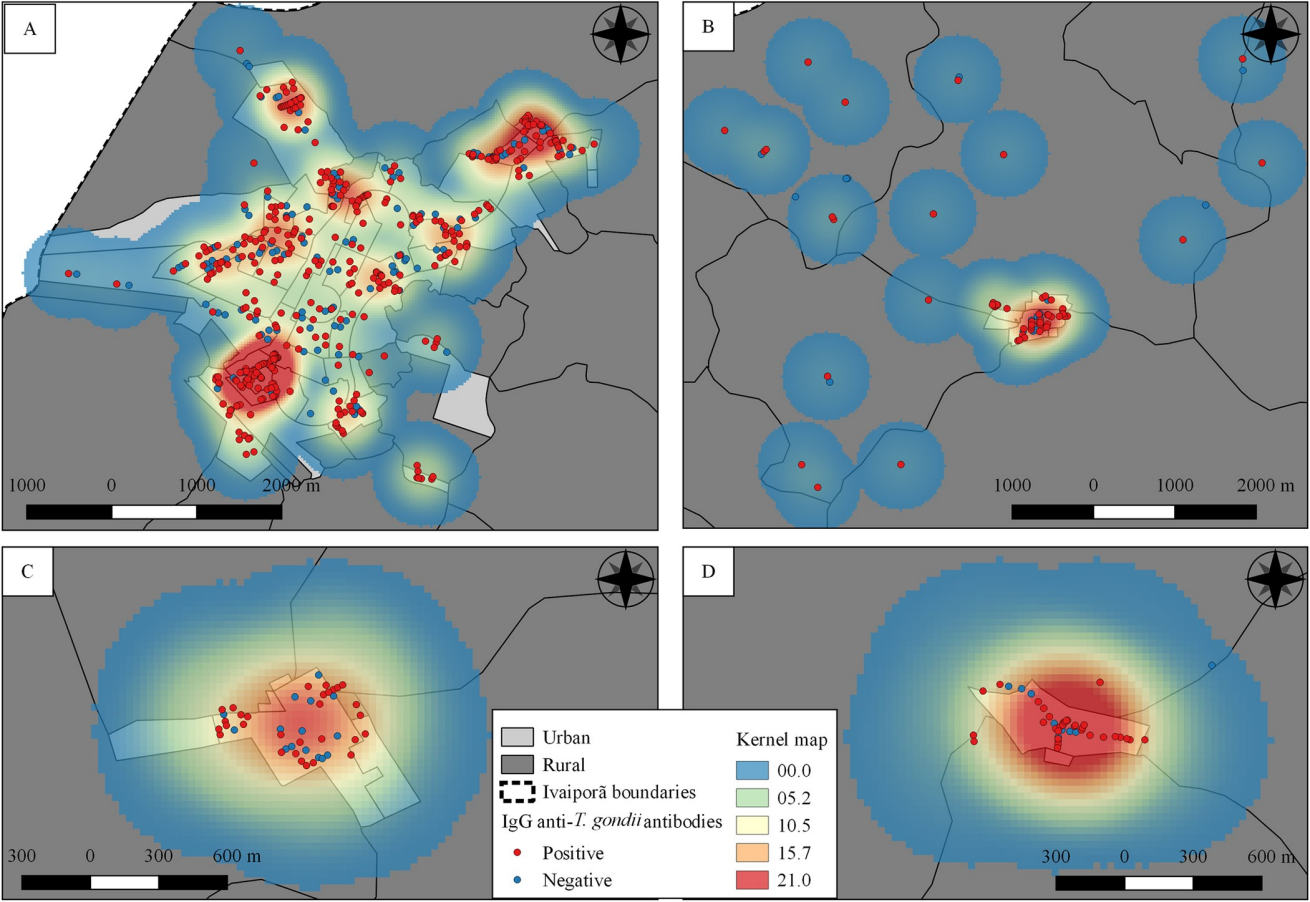
Kernel map of the seropositivity results for IgG anti-T. gondii antibodies in 715 human samples tested by IFAT, from 2015 to 2016 in the city of Ivaiporã, Paraná State, Brazil”. Letters represent the four municipal districts: A: Ivaiporã; B: Jacutinga; C: Alto Porã; D: Santa Bárbara.

Spatial analysis has shown no significant cluster on studied area. However, most heat areas of Kernel map has shown occurrence of higher intensity number of seropositive individuals concentrated on peri-urban areas (Fig 2).

## Discussion

Prevalence of anti-*T*. *gondii* on vulnerable population herein (73.57%) has been higher than 41.54% on northern-central [25], 213/356 (59.8%) general population people and 40/66 (60.6%) pregnant women of northern [28] Paraná State, but similar to 527/599 (87.97%) elderly people from Rio Grande do Sul State [29]. Prevalence was expected similar to overall 196/280 (70.00%) seropositivity with 143/207 (69.08%) in rural and 53/73 (72.60%) in urban areas of a slum community about 100 miles from Ivaiporã [19] due to similarities in climate, population dynamics and definitive / intermediate (preys) hosts [30].

The epidemiological profile of higher risk of seropositivity for *T*. *gondii* was characterized by more years of life, fewer years of study, lower family income, no occupation outside home and living in household lacking water tank. Thus, risk factors herein were more likely associated to socioeconomic conditions than well-established alimentary habits such as consumption of raw or undercooked meat [4], unwashed vegetables [31] and cat ownership and soil handling [32,33]. In addition, spatial distribution of seropositive cases has demonstrated a higher toxoplasmosis occurrence in suburban than central city areas, where mostly low-income communities have been located.

The odds ratio of adult and elderly seropositivity to IgG anti-*T*. *gondii* antibodies were respectively 2.60 and 3.10 higher than in people aged 18 years old or younger. Such association have probably occurred due to longer risk exposure, increasing the individual chances of been infected over time [29,34,35]. The disease manifestation may not be related to latent stage but the loss of disability-adjusted life years (DALY) has been suggested on long term [10]. Previous studies have indicated an association of seropositivity with psychiatric disorders such as bipolarity, obsessive-compulsive disorder, schizophrenia, suicide attempting, compromised memory in elderly people and Parkinson [8,36,37].

On the other hand, the seronegative results of younger and adolescent populations should be considered on social risk of adolescence pregnancy, with higher chances of primo-infection by *T*. *gondii* on beginning of woman fertile period. Transplacental transmission of parasite to fetus may trigger the development of serious congenital damages and sequels over the years [6,38]. A cohort study on congenital toxoplasmosis has shown that lower socioeconomic level was a risk factor for non-screening participation during pregnancy, resulting in higher likelihood on disease onset through pregnancy and neonatal in low-income mothers; disease risk was reduced in pregnant women with higher socioeconomic level [39].

Low level of school years was observed in high prevalence of IgG anti-*T*. *gondii* antibodies herein, with illiteracy associated to 4.37-fold higher odds ratio for IgG anti-*T*. *gondii* antibodies. Lack of information about potential transmission ways may have exposed vulnerable populations to infection [40], and may indicate the importance of education as health promotion and disease prevention. School years and toxoplasmosis were statistically associated (p = 0.010) in a survey with 712 pregnant women from northeastern and 229 (p = 0.049; OR:2.52) from central-western Brazil [34,35], highlighting that prevalence may be higher in illiterate women. As expected, education in public health for pregnant women has shown to be an effective prevention measure [41]. In addition, detection of such variable obtained herein in non-pregnant population may suggest similar results in all populational extracts, supporting educational programs as public health strategy for toxoplasmosis prevention.

Lacking of household water tank was statistically associated to seropositivity for anti-*T*. *gondii* antibodies, and previously recognized by the Paraná state as an indicator of social vulnerability as mirror of low-income status, linked to lack of resources for basic sanitary conditions [22]. However, presence of water tank without adequate seal or maintenance may fail to prevent felid feces contamination, as oocysts may environmentally survive for years [42]. Not surprisingly, the biggest world outbreak of toxoplasmosis was reported in nearby northern Parana State in 2001, with the city water reservoir as the source for oocyst spreading and infection of at least 426 inhabitants [43].

Lower socioeconomic level has been associated to *T*. *gondii* infection due to non-treated and non-filtrated drinking water as the main risk factor for low-income populations [44]. Despite hand and food washing have been associated to toxoplasmosis prevention [5], hygiene habits were assessed only by self-evaluation questionnaire and therefore may have been overestimated in the present study. Regardless, occupation classified by retired, unemployed people and housekeeping individuals herein were more likely to present anti-*T*. *gondii* antibodies (p<0.001), clearly demonstrating that *T*. *gondii* exposure has been influenced by socioeconomic issues.

It should be noted that even with an adequate sample size, low number of negatives (189/715) can affect the results when subcategory analysis are performed. For example, the categories of age (above 60 years old) and schooling (illiterate) showed wider confidence intervals and some variables such as meat consumption and garbage disposal showed wider confidence intervals but non-significant association with toxoplasmosis.

Spatial distribution of individuals with anti-*T*. *gondii* antibodies has shown two areas with high density of points in the Kernel map, one with the lowest reported family income and the other with unreported income due to recent invasion settlement. Nonetheless, both areas were characterized by inadequate constructions and lack of potable water supply. A previous study in a bigger city within the same northern state region has shown one area only of cluster, which was also correlated to low-income families [25]. In a rural area of northern France [45] and an urban area of northeastern Mainland China [46], despite the spatial analysis approach of soil samples has shown environmental contamination of *T*. *gondii* oocysts, no socioeconomic aspects or vulnerability associated risks for infection were tested. In the present study, vulnerability indicators have been directly associated to presence of anti-*T*. *gondii* antibodies.

Differences on seroprevalence found herein may be associated to socioeconomic conditions, which may influence on alimentary and hygiene habits [47,48]. Moreover, vulnerability as a group of risk factors may be a major common denominator which has a daily impact on individual exposure to pathogens [40]. In such scenario, epidemiology of zoonotic diseases may demand a comprehensive and holistic approach, denominated as One Health and contemplating human, animal and environmental health [48].

Finally, to the author’s knowledge, no study has yet attempted to assess social vulnerability, either alone or in a multi-factorial investigation, and its association to *T*. *gondii* infection. Surprisingly, toxoplasmosis has already been ranked as the second among foodborne parasitic diseases, particularly in vulnerable populations [49].

## Conclusions

Socioeconomic vulnerability was statistically associated to *Toxoplasma gondii* exposure, which included adult and elderly ages, illiteracy, no occupation (retired, unemployed and own housekeeping people) and lack of household water tank were demonstrated by multivariate analysis; influence of low-income family was demonstrated by spatial analysis. On the other hand, variables well established as associated risk factors for toxoplasmosis as living area, yard hygiene, meat ingestion and pet ownership were not statistically significant in the multivariate model and may play a secondary role in such communities.

## Supporting information

S1 File Questionnaire used to by the department of Preventive Veterinary Medicine of the Londrina State University to collect epidemiological data on social, economic and environmental factors. (DOCX)
