# Supplementary material for: Socioeconomic vulnerability associated to Toxoplasma gondii exposure in southern Brazil
Source: PLoS One. 2019 Feb 14;14(2):e0212375. doi: 10.1371/journal.pone.0212375 (PMC6375698; doi:10.1371/journal.pone.0212375)
Supplement: S1 File — (DOCX) [file pone.0212375.s001.docx]

**Supporting information 1:** Questionnaire used to by the Department of Preventive Veterinary Medicine of the Londrina State University to collect epidemiological data on social, economic and environmental factors.

| **SOCIALECONOMIC** | | | | | | | | | | | | | | | | | | | | | | | | | | |
| --- | --- | --- | --- | --- | --- | --- | --- | --- | --- | --- | --- | --- | --- | --- | --- | --- | --- | --- | --- | --- | --- | --- | --- | --- | --- | --- |
| Interview date: | | | | | | | | | | | | | | | | Registration in SUS ( )1. Yes ( ) 2. No | | | | | | | | | | |
| Name of the Volunteer: | | | | | | | | | | | | | | | |  | | | | | | | | | | |
| Name of guardian (in case of under 18 years of age): | | | | | | | | | | | | | | | | | | | | | | | | | | |
| Street/Nº: | | | | | | | | | | | | | | | | | | | | | | | | | Neighborhood: | |
| Gender: (xxx)1. F (xxx)2. M | Age: | | | | | | | Occupation: | | | | | | | | | | | | | | | | | | |
| Telephone: | | | | | | | | | | | | | | | | | | | | | | | | | | |
| Family income monthly: (R$ ___________________)  (xxx)1. Up to 1 MW (Minimum Wage) (xxx)2. Between 1 to 3 MW (xxx)3. More than 3 MW | | | | | | | | | | | | | | | | | | | | | | | | | | |
| Schooling: | | | | | | | | | | | | | | | | | | | | | | | | | | |
| **ENVIRONMENTAL** | | | | | | | | | | | | | | | | | | | | | | | | | | |
| How much time living in this address? | | | | | | | | | | | | | | | | | | How many people share the house? | | | | | | | | |
| Source of Potable water? (xxx) 1. Public (xxx) 2. Well (xxx) 3. Spring water | | | | | | | | | | | | | | | | | | | | | | | | | | |
| When raining, there is water accumulated near home? (xxx) 1. Yes (xxx) 2. No | | | | | | | | | | | | | | | | | | | | | | | | | | |
| There is water tank in the house? | | (xxx) 1. Yes | | | | | | | | | | | | | | | | | | (xxx) 2. No | | | | | | |
| The water tank is closed? | | (xxx) 1. Yes | | | | | | | | | | | | | | | | | | (xxx) 2. No | | | | | | |
| What frequency the water tank is cleaned? | | (xxx) 1. One time in 6 months | | | | | | | | | | | | | | | | | | (xxx) 2. One time per year | | | | | | |
|  | | (xxx) 3. Don’t clean | | | | | | | | | | | | | | | | | | (xxx) 4. Don’t know the frequency | | | | | | |
| How is the disposal of wastewater? | | (xxx) 1. Public | | | | | | | | | | | | | (xxx) 2. Cesspit | | | | | | | | (xxx) 3. Don’t have disposal | | | |
| How is the garbage disposal? | | | | | | (xxx) 1. Plastic bags | | | | | | | | | | | | | | | | | (xxx) 2. Open Litter | | | |
| (xxx) 3. Plastic bag in litter | | | | | | (xxx) 4. Burn | | | | | | | | | | | | | | | | | (xxx) 5. Open air | | | |
| There is unoccupied areas near house? (xxx) 1. Yes (xxx) 2.No | | | | | | | | | | | | | | | | | | | | | | | | | | |
| There is a forest near the residence? (xxx) 1. Yes (xxx) 2.No | | | | | | | | | | | | | | | | | | | | | | | | | | |
| Do you have a private bathroom with running water? (xxx)1. Yes (xxx)2. No | | | | | | | | | | | | | | | | | | | | | | | | | | |
| There is restroom outside the house? (xxx) 1. Yes (xxx) 2.No | | | | | | | | | | | | | | | | | | | | | | | | | | |
| Which frequency the garden is cleaned? (xxx) 1. Daily (xxx) 2. Weekly (xxx) 3. Biweekly  (xxx) 4. Monthly (xxx) 5. Don’t do | | | | | | | | | | | | | | | | | | | | | | | | | | |
| The garden has: (places that can accumulate rodents, mosquitoes, among others)  (xxx)1. Garbage (xxx) 2. Tree leaves (xxx) 3. Construction debris (xxx)4. Cleaned | | | | | | | | | | | | | | | | | | | | | | | | | | |
| Have you ever seen rats in the house? | | (xxx) 1. Yes, inside the house | | | | | | | | | | | | | | | | | | | (xxx) 2. Yes, in the garden (xxx)3. No | | | | | |
| What do you use to control rats? (xxx)1. Cats | | | | | | | | | (xxx)2. Poison (xxx)3. Mousetrap (xxx)4. Nothing | | | | | | | | | | | | | | | | | |
| Have you ever seen ticks in the house? (xxx)1. Yes, in the backyard  (xxx)2. Yes, inside the house (xxx)3. No | | | | | | | | | | | | | | | | | | | | | | | | | | |
| Has mosquitoes in the house? (xxx)1. Few (xxx)2. A lot (xxx) Don’t | | | | | | | | | | | | | | | | | | | | | | | | | | |
| How do you control mosquitoes? | | (xxx)1. Net in the window | | | | | | | | | | | | | | | | | | | | (xxx)2. Repellent in the body | | | | |
|  | | (xxx)3. Repellent in the environment | | | | | | | | | | | | | | | | | | | | (xxx)4. Don’t control | | | | |
| Have you seen wild animals near your residence? | | | | | | | | | | | | | (xxx)1. Yes (xxx) 2. No | | | | | | | | | | | | | |
| If so, which one? (xxx)1. Skunk (xxx)2. Capybara (xxx)3. Monkey (xxx) 4.Others (xxx) 5. Bat | | | | | | | | | | | | | | | | | | | | | | | | | | |
|  | | | | | | | | | | | | | | | | | | | | | | | | | | |
|  | | | | | | | | | | | | | | | | | | | | | | | | | | |
| **INDIVIDUAL** | | | | | | | | | | | | | | | | | | | | | | | | | | |
| Do you clean fruits and vegetables before eat? | | | | | | | | | | | | | | | | | | | | | |  | | | | |
| (xxx)1. Yes, always | | | | (xxx)2. Yes, almost always | | | | | | | | | | | | | | | | | | (xxx)3. No | | | | |
| What do you use to clean? | | | | | | | | | | | | | | | | | | | | | |  | | | | |
| (xxx)1. Only water | | | | (xxx) 2. Bleach | | | | | | | | | | | | | | | | | | (xxx) 3. Vinegar | | | | |
| Do you wash your hands before eat? | | | | | | | | | | | | | | | | | | | | | |  | | | | |
| (xxx)1. Always, with water and soap  (xxx)2. Almost always, with water and soap | | | | | | | | | | | (xxx)3. Almost always, water  (xxx)4. Sometimes | | | | | | | | | | | | | | | (xxx) 5. No |
| Do you wash your hands after go to the restroom? | | | | | | | | | | | | | | | | | | | | | | | | | | |
| (xxx)1. Always, with water and soap  (xxx)2. Almost always, with water and soap | | | | | | | | | | | (xxx)3. Almost always, water  (xxx)4. Sometimes | | | | | | | | | | | | | | | (xxx) 5. No |
| What kind of meat do you eat? (xxx)1. Beef (xxx)2. Pork (xxx) 3. Lamb (xxx) 4. Poultry  (xxx)5. Fish (xxx)6. Don’t eat meat | | | | | | | | | | | | | | | | | | | | | | | | | | |
| Do you have the habit to eat: | | | | | | | | | | | | | | | | | | | | | | | | | | |
| Do you eat meat raw or undercooked? | | | | (xxx)1. Yes | | | | | | | | | | | | | | |  | | | (xxx)2. No | | | | |
| Raw kebab | | | | (xxx)1. Yes | | | | | | | | | | | | | | | | | | (xxx)2. No | | | | |
| Barbecue undercooked | | | | (xxx)1. Yes | | | | | | | | | | | | | | | | | | (xxx)2. No | | | | |
| Smoked sausage | | | | (xxx)1. Yes | | | | | | | | | | | | | | | | | | (xxx)2. No | | | | |
| Fresh sausage | | | | (xxx)1. Yes | | | | | | | | | | | | | | | | | | (xxx)2. No | | | | |
| Homemade salami | | | | (xxx)1. Yes | | | | | | | | | | | | | | | | | | (xxx)2. No | | | | |
| Raw milk? | | | | (xxx)1. Yes | | | | | | | | | | | | | | | | | | (xxx)2. No | | | | |
| Do you have frequent contact with soil or sand? | | | | | | | | | | | | | (xxx)1. Yes | | | | | | | | | | | | (xxx)2. No | |
| Do you visit forest areas? | | | | (xxx)1. Yes, every day | | | | | | | | | | | | | | | | | | (xxx)2. Yes, one time per week | | | | |
| (xxx)3. Yes, one time per month | | | | (xxx)4. Yes, one time per year | | | | | | | | | | | | | | | | | | (xxx)5. No | | | | |
| Have you ever see ticks in your body? | | | | | | | | | | | | | | (xxx)1. Yes | | | | | | | | (xxx)2. No | | | | |
| Were you hospitalized last year because of illness? | | | | | | | | | | | | | | | | (xxx)1. Yes | | | | | | (xxx)2. No | | | | |
| Do you know what disease was? (xxx)1. Yes (xxx)2. No What? | | | | | | | | | | | | | | | | | | | | | | | | | | |
| Do you have diarrhea during last week? | | | | | | | | | | | | | | | | (xxx)1. Yes | | | | | | (xxx)2. No | | | | |
| Have you ever had hepatitis or jaundice? (xxx)1. Yes (xxx)2. No (xxx)3. Don’t know | | | | | | | | | | | | | | | | | | | | | | | | | | |
| Do you have any lesion in skin? | | | | (xxx)1. Yes (xxx)2. No | | | | | | | | | | | | | | | | | | | | Local: | | |
| How many time do you have the lesion? | | | | | | |  | | |  | | | | | | | | | | | | | | | | |
| What kind in this lesion? | | | (xxx)1. Nodular (xxx)2. Ulcerate | | | | | | | | | | | | | | | | | | | | |  | | |
| Have you traveled in last 12 months? | | | | | (xxx)1. Yes | | | | | | | | | | | | (xxx)2. No | | | | | | | | | |
| Which destination? | | |  | | | | | | | | | | | | | | | | | | | | |  | | |
| How many dogs and cats are in the house? | | | | | | | | | | | | Dog: Cat: ( xxx) No | | | | | | | | | | | | | | |
